# Mallory-Weiss Syndrome Triggered by Undigested Food Residues in a 90-Year-Old Male: An Unusual Etiology

**DOI:** 10.7759/cureus.94114

**Published:** 2025-10-08

**Authors:** Shashwat Arora, Ishita Singh, Waqas Alauddin, Prabhnoor Monga, Shreyash Yadav

**Affiliations:** 1 Medicine, Naraina Medical College & Research Centre, Kanpur, IND; 2 Medicine, Lala Lajpat Rai Memorial Medical College, Meerut, IND; 3 Physiology, Naraina Medical College & Research Centre, Kanpur, IND; 4 Medicine, Hamdard Institute of Medical Sciences and Research, New Delhi, IND

**Keywords:** endoscopy, hematemesis, hemoclip, mallory-weiss tear, upper gastrointestinal bleed

## Abstract

Mallory-Weiss syndrome is an uncommon cause of upper gastrointestinal bleeding, typically resulting from mucosal lacerations induced by retching, vomiting, or coughing. Although well described in middle-aged individuals, its occurrence in elderly patients is unusual and may present atypically. We report the case of a 90-year-old male who developed hematemesis following vomiting undigested food residues, without preceding forceful emesis. Endoscopy revealed a Mallory-Weiss tear with active bleeding, successfully treated with hemoclips, and an incidental duodenal polyp. This case underscores the role of non-classical triggers in elderly patients and highlights the importance of prompt endoscopic evaluation and therapy.

## Introduction

Mallory-Weiss syndrome (MWS) is defined by longitudinal mucosal tears at the gastroesophageal junction and represents approximately 5-15% of non-variceal upper gastrointestinal hemorrhage [1]. It is classically associated with repeated retching, vomiting, or coughing, which lead to abrupt elevations in intra-abdominal pressure, leading to mucosal disruption [2]. While MWS is more commonly encountered in middle-aged populations, its presentation in the elderly is infrequent and often atypical [2]. Compared with younger adults, cases in older patients are rarely described, but when they occur, they frequently exhibit unusual clinical manifestations and a greater degree of hemodynamic instability [3]. Age-related physiological changes, including impaired gastric motility, autonomic dysfunction, and delayed mucosal healing, may predispose elderly individuals to mucosal injury even without forceful emesis [2,4]. These vulnerabilities allow relatively minor or uncommon mechanical insults, such as swallowing or regurgitating incompletely digested food, to precipitate significant bleeding [5].

Emerging evidence highlights additional non-classical precipitants, including bezoars, dialysis-associated physiological alterations, or trauma induced by retained gastric contents, broadening the spectrum of MWS pathogenesis [6]. Here, we report a rare case of MWS triggered by undigested food residues in a 90-year-old man, underscoring an atypical etiology and the therapeutic value of endoscopic intervention.

## Case presentation

A 90-year-old male with a medical history of type 2 diabetes mellitus, hypertension, and occasional alcohol intake presented to the emergency department with hematemesis. He described vomiting undigested food consumed the previous evening, including bitter gourd and carrot, without antecedent retching or forceful emesis. On examination, he was pale with mild pedal edema. Supine blood pressure was 100/75 mmHg, dropping to 88/68 mmHg on standing. His pulse rate was 76 beats per minute in the supine position, and oxygen saturation was 97% on room air. Cardiovascular evaluation demonstrated T-wave inversions on ECG.

Laboratory investigations revealed reduced hemoglobin, elevated blood urea nitrogen, and elevated creatinine. The detailed laboratory parameters with corresponding reference ranges are summarized in Table 1.

**Table 1:** Laboratory investigations of the patient at presentation and during hospitalization. The table demonstrates the laboratory profile of the patient at admission and during follow-up. The trend reflects recovery of renal parameters and hemoglobin stabilization following intervention.

Parameter	At presentation	Follow-up (day 3)	Reference range	Interpretation
Hemoglobin (g/dL)	8.8	10.5	13.0–17.0	Decreased
Blood urea nitrogen (mg/dL)	47	32	7–20	Increased
Serum creatinine (mg/dL)	1.9	1.4	0.7–1.3	Increased
Random glucose (mg/dL)	196	150	70–140	Increased

**Figure 1:**
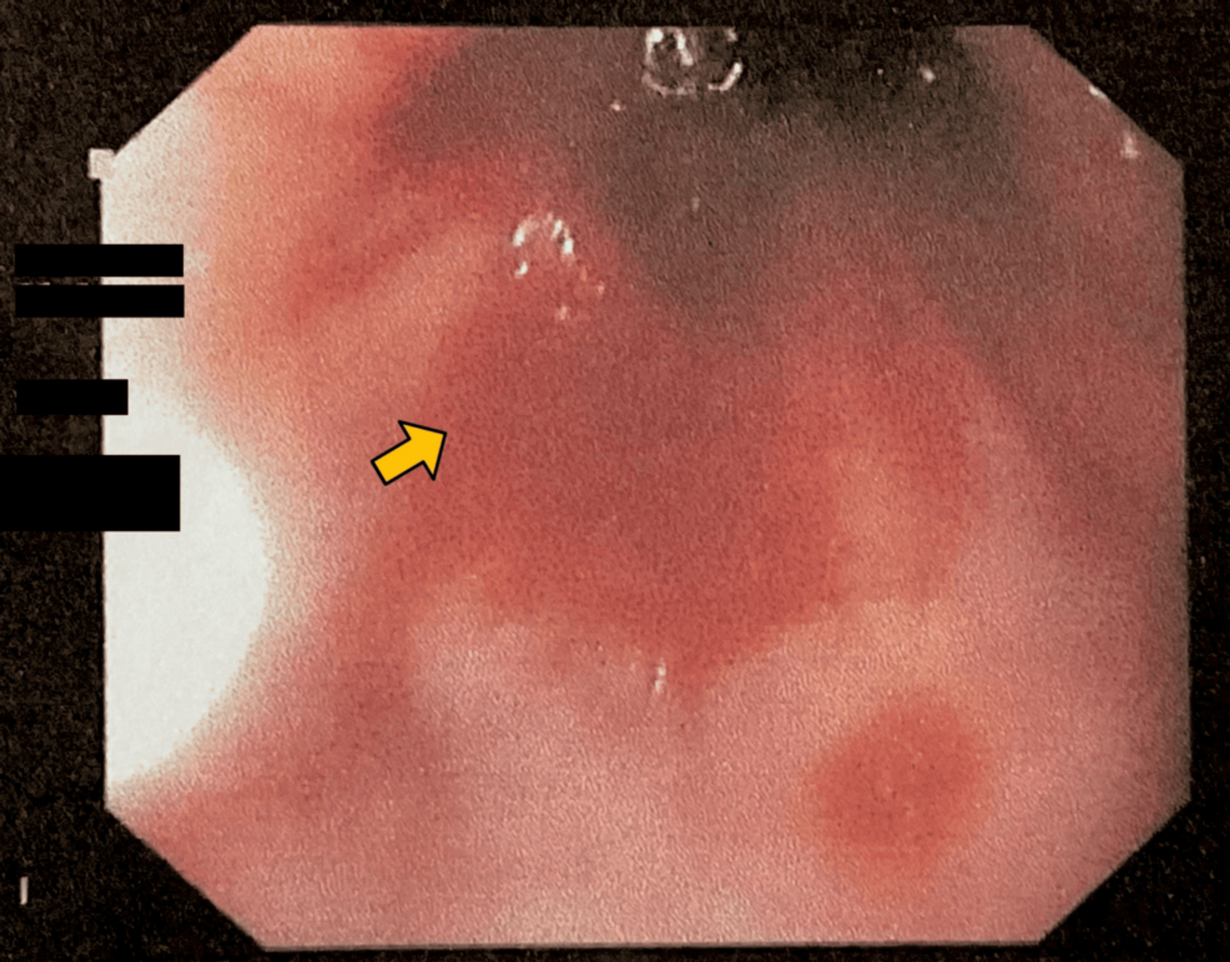
Endoscopic view of the site of the tear with blood oozing.

**Figure 2:**
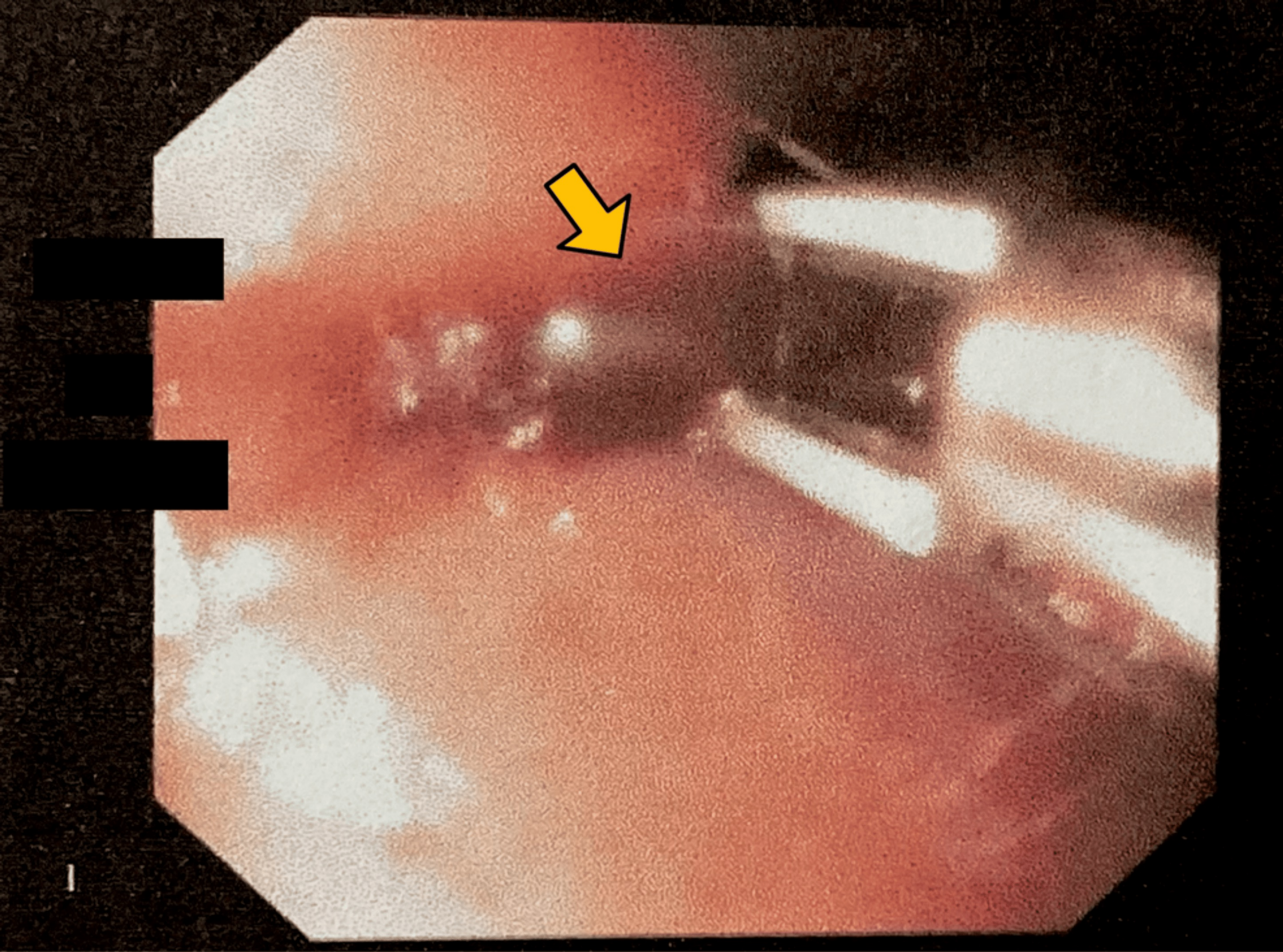
Site of the first hemoclip.

**Figure 3:**
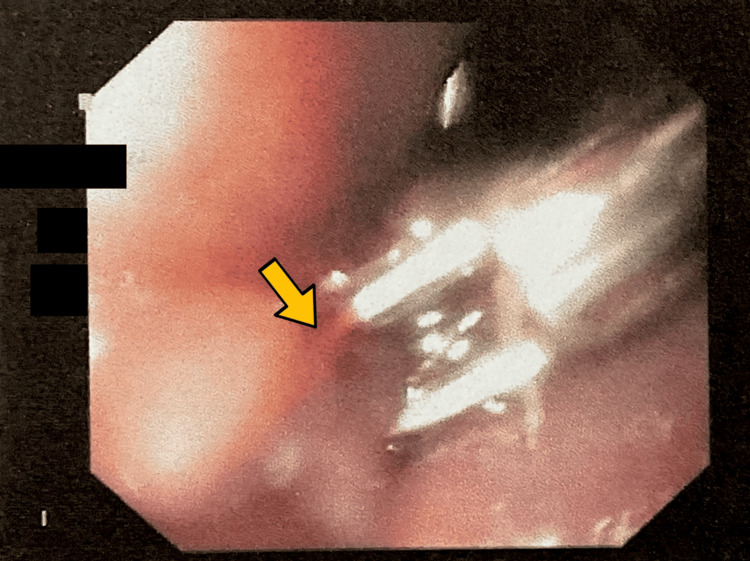
Site of the second hemoclip.

**Figure 4:**
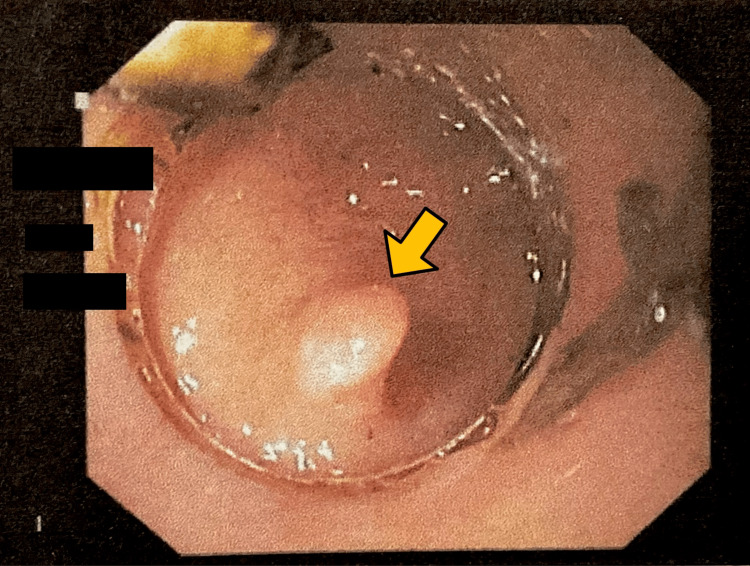
Incidental duodenal polyp seen in the first part of the duodenum.

Endoscopy revealed a longitudinal Mallory-Weiss tear at the gastroesophageal junction measuring 1 × 0.5 mm with active bleeding (Figure 1). Hemostasis was successfully achieved with the application of the first hemoclip (Figure 2) and the second hemoclip (Figure 3). An incidental duodenal polyp was also identified, excised, and biopsied (Figure 4). All images were anonymized to obscure patient-identifying information.

The patient was managed with intravenous proton pump inhibitor infusion, fluid resuscitation, discontinuation of antihypertensive medications, and optimization of diabetes management with gliptins and basal insulin. His condition stabilized following treatment, and he was discharged in stable condition on the fourth postoperative day.

## Discussion

Although MWS is a recognized contributor to upper gastrointestinal bleeding, its prevalence among elderly patients remains lower than in younger cohorts [1,2]. The conventional mechanism involves a sudden rise in intra-abdominal pressure secondary to retching or vomiting; however, less typical triggers are increasingly reported, particularly in frail or older populations [2].

In the present case, the injury was most plausibly due to direct trauma from undigested food residues rather than repetitive vomiting. Advanced age is linked to delayed gastric emptying [2], diabetic autonomic neuropathy [4], and reduced mucosal integrity, factors that collectively heighten susceptibility to mucosal tears from relatively minor mechanical stressors [7]. Yin et al. [7] observed that MWS in elderly patients often manifests with more severe bleeding and marked hemodynamic compromise, even in the absence of vigorous vomiting. Other published reports have described atypical associations such as MWS in patients undergoing hemodialysis [8] and cases precipitated by bezoars [9]. The clinical features in our patient parallel these unusual presentations, with undigested food residues serving as the inciting factor.

Endoscopic therapy remains the cornerstone of management. While epinephrine injection was historically the primary modality, recent evidence favors mechanical techniques such as hemoclipping, which demonstrate superior hemostasis and reduced risk of rebleeding [10]. In our patient, bleeding was effectively controlled with endoscopic hemoclips. Additionally, the incidental detection of a duodenal polyp during the same procedure reinforces the importance of comprehensive endoscopic assessment, though the polyp itself was unrelated to the acute bleeding episode [10].

This case highlights that in elderly individuals, even seemingly trivial mechanical factors can precipitate life-threatening upper gastrointestinal hemorrhage. Early recognition and timely endoscopic intervention remain essential for achieving favorable clinical outcomes [11].

## Conclusions

We report a rare case of MWS in a 90-year-old man, precipitated by undigested food residues rather than the typical triggers of retching or forceful vomiting. This case underscores that in older patients, atypical and relatively minor mechanical insults may result in severe upper gastrointestinal bleeding, with clinical features differing from the classical pattern. Given the high burden of comorbidities and increased complication risk in the elderly, hematemesis in this population should be approached with a broad differential diagnosis, extending beyond peptic ulcer disease or variceal bleeding. Early endoscopic examination plays a dual role, both confirming the diagnosis and enabling immediate therapeutic intervention. Awareness of such atypical presentations is vital to guide prompt management, reduce complications, and improve outcomes in vulnerable elderly patients.
